# Influence of Temperature on Hyperelastic Mechanical Behavior of Accelerated Aged EPDM Rubber

**DOI:** 10.3390/polym17121626

**Published:** 2025-06-11

**Authors:** Zhaonan Xie, Dong Jia, Xicheng Huang, Kai Zhang, Shunping Yan, Junhong Chen, Jiaxing Li, Weizhou Zhong

**Affiliations:** 1 Institute of Systems Engineering, China Academy of Engineering Physics, Mianyang 621999, China; 2 Shock and Vibration of Engineering Materials and Structures Key Laboratory of Sichuan Province, Mianyang 621999, China

**Keywords:** EPDM rubber, accelerated aging, constitutive model, hyperelastic behavior, temperature influence

## Abstract

EPDM (Ethylene Propylene Diene Monomer) rubber is a crucial engineering material, and its mechanical behavior changes with aging duration and ambient temperature. The effects of temperature on the hyperelastic behavior of unaged and aged EPDM rubber are investigated by conducting accelerated aging tests under constant compression and uniaxial compression tests at different temperatures. The experimental results show that prolonged aging induces EPDM rubber to exhibit an approximately linear hardening trend under a constant temperature. For aged EPDM rubber, its stiffness initially decreases and then increases with test temperature. The stress hardening factor was introduced to characterize the influence of the test temperature on the aging effect. The factor exhibits a decreasing trend and then an increasing trend with respect to compression test temperature. The curve of the stress hardening factor versus temperature is approximately a quadratic function. To fit the results, a Neo–Hooke model, a Mooney–Rivlin model, and an improved Mooney–Rivlin model were tested for their fit with the EPDM rubber compression data, covering different experimental conditions. The improved Mooney–Rivlin model had the most consistent results with the experimental data. Based on the experimental results, the parameters of the improved Mooney–Rivlin model were extended to model the effects of temperature and aging time. The proposed constitutive model can effectively describe the hyperelastic behavior of aged EPDM rubber tested at different temperatures.

## 1. Introduction

EPDM (Ethylene Propylene Diene Monomer) rubber exhibits excellent properties including insulation, heat resistance, chemical stability, and resilience. It is widely used in the tire industry [1], thermal insulation [2,3], mechanical sealing [4,5], and vibration isolation [6]. In practical applications, the service environment of EPDM products is usually not maintained at a constant temperature. For example, sealing rubber components undergo seasonal temperature variation. Cyclic loading induces rubber to generate self-heating due to its viscoelastic nature. It is crucial to analyze the temperature effect on the mechanical behavior of EPDM rubber.

The temperature-dependent mechanical behavior of EPDM rubber is also influenced by thermo-oxidative aging. EPDM products are exposed to thermo-oxidative environments for long periods, inevitably undergoing oxidative reactions. Thermo-oxidative aging brings about irreversible changes in the microstructure of EPDM rubber, such as chain crosslinking and scission, the formation of oxidative groups, and the migration of additives [7,8,9]. These microstructural transformations significantly affect the macroscopic thermomechanical response of EPDM rubber under different temperature conditions. Therefore, developing a theoretical model that can explicitly describe the influence of temperature effects and thermo-oxidative aging on the mechanical behavior of EPDM rubber is an important challenge.

The nonlinear characteristics of rubber under large deformations are usually described using hyperelastic constitutive models. Many hyperelastic constitutive models have been developed, which can be generally classified into two types: phenomenological models [10,11,12] and molecular network-based models [13,14,15]. Many researchers [16,17,18,19] have systematically analyzed the advantages and limitations of different hyperelastic models, providing valuable references for selecting appropriate models. However, conventional hyperelastic models focus on the behavior of unaged rubber at a single temperature and are unable to describe the effects of temperature and thermo-oxidative aging.

In recent years, researchers have investigated hyperelastic constitutive models of rubber materials, which incorporate temperature and thermo-oxidative aging effects. For the incorporation of temperature effects, some studies [20,21,22,23] have proposed thermodynamic models for rubber materials by decomposing the deformation gradient into a thermal expansion part and an “effective” mechanical deformation part by the assumption of thermomechanical decomposition. Based on the temperature-dependent mechanical testing of rubber materials, some researchers [24,25,26,27,28,29] have linked the parameters of hyperelastic models to temperature. Rodas [30] and Li [31] incorporated the influences of temperature and carbon black fillers into hyperelastic models. Jiang [32] extended the five-parameter Mooney–Rivlin model by introducing a multiplicative decomposition framework to incorporate temperature and strain-rate effects. Yan [33] analyzed the influence of temperature on the compressive behavior of poly methyl-vinyl silicone rubber foams exposed to different radiation doses and extended the Ogden Hyperfoam model parameters to include temperature and radiation effects. For aging effects, Korba [34] proposed empirical functions based on experimental data to describe the evolution of model parameters with aging temperature and time within their weighted-function-based (WFB) hyperelastic model. The effects of thermo-oxidative aging on hyperelastic models were described using the Arrhenius relationship [35,36,37,38,39,40]. Shakiba [41] and Zhi [42] established the evolution of Arruda–Boyce model parameters with aging time by considering changes in crosslink density. Li [43] and Du [44] introduced aging characteristic functions to represent the aging effects of hydroxyl-terminated polybutadiene and expanded the hyperelastic model using a multiplicative decomposition approach. Some researchers [45,46] found that model parameters exhibit a linear trend with aging time.

Many studies focus on the hyperelastic behavior of unaged rubber at different temperatures and aged rubber at room temperature. Few studies focus on hyperelastic constitutive models of aged rubber materials at different temperatures, which makes it difficult to meet current production and design requirements. This study aims to investigate the effects of temperature on the hyperelastic mechanical behavior of aged EPDM rubber. Firstly, accelerated aging tests of EPDM rubber were conducted at an elevated temperature and under a constant compressive strain condition. Then, the variations in the compression stress–strain curves of unaged and aged EPDM rubber at different temperatures were analyzed. Finally, an improved Mooney–Rivlin model was proposed, which extends the Mooney–Rivlin model to a three-term form and incorporates the effects of aging and temperature. The evaluation results show that the prediction results of this model are in good agreement with the experimental data. This study can help estimate the long-term service performance and lifetime of rubber products under variable temperature conditions.

## 2. Materials and Methods

### 2.1. Material and Specimen

**Figure 1:**
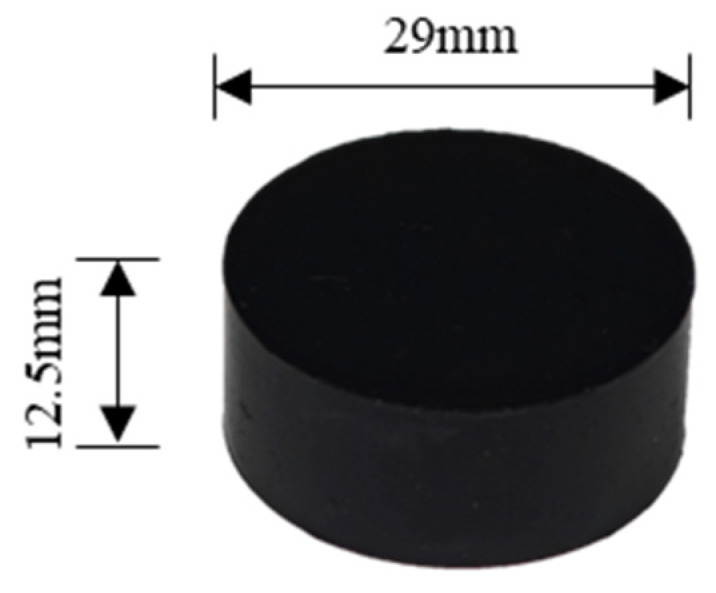
The EPDM rubber compression specimen.

The EPDM rubber used in this study was prepared based on the formulation proposed by Huang [47], and the main components are listed in Table 1. All ingredients used in the formulation were of industrial grade and commercially available. The preparation process is as follows: EPDM, organo-modified montmorillonite (OMMT), and the compatibilizer EPDM-g-MAH were melt-compounded at 110 rpm, 90 °C for 15 min to achieve intercalation. Then the compound was transferred to a two-roll mill. The rolls were heated to 60 °C, and the material was passed repeatedly through a narrow roll gap to form a continuous sheet. The roll gap was widened, and the rolls were heated to 80 °C. Carbon black, ZnO, stearic acid, and antioxidants were added sequentially and uniformly mixed to obtain Compound I, which was then naturally cooled. Subsequently, the vulcanizing agent and accelerator were incorporated into Compound I to obtain Compound II. Compound II was then poured into the designed mold to form cylindrical samples for compression. The molds were placed in a compression molding machine and cured at 160 °C under 15 MPa for 1 h to obtain cylindrical specimens, as shown in Figure 1.

### 2.2. Accelerated Aging Test and Time–Temperature Equivalent Principle

**Figure 2:**
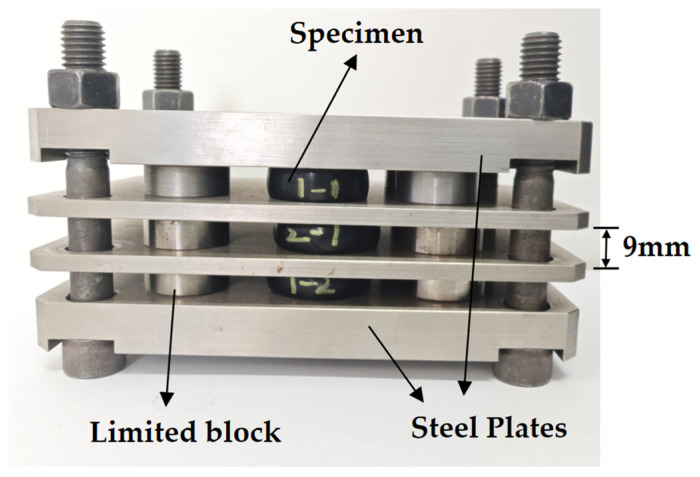
Pre-compression device.

EPDM rubber products usually undergo aging in a deformed state during the service process. A pre-deformation device was applied in this study, as shown in Figure 2. The device applies compression to the EPDM sample by tightening bolts on the pressure plates, while the compression ratio is controlled using a limit block. In this setup, a limit block with a height of 9 mm was used, corresponding to a constant compression ratio of 28%. As EPDM rubber ages very slowly at room temperature, accelerated aging was performed by the pre-compressed specimens in an air-circulated oven. The oven temperature was set to 393 K. Based on the Arrhenius equation and time–temperature equivalence principles [48,49,50,51,52], the aging time at 393 K can be equivalently converted to the aging time at room temperature. The conversion equation is as follows:

(1) tRetT=expEaR1393−1T

where *t_Re_* denotes the aging time at the reference temperature, *t_T_* is the aging time at room temperature *T*(K), *E_a_* is the activation energy, and *R* is the universal gas constant (8.314 J/(K·mol)).

Considering seasonal variations in room temperature, the room temperature throughout the year is approximated as 283 K in spring, 313 K in summer, 293 K in autumn, and 273 K in winter. Accordingly, one year of natural aging is represented by a combination of 3 months of aging at each of these temperatures: 273 K, 283 K, 293 K, and 313 K. To equivalently obtain EPDM samples aged at room temperature for 7 years, 9 years, and 13 years, the actual test time was calculated by Equation (1). After the accelerated aging test was completed, EPDM specimens were placed at room temperature to recover until the height remained unchanged.

### 2.3. Temperature-Dependent Uniaxial Compression Test

**Figure 3:**
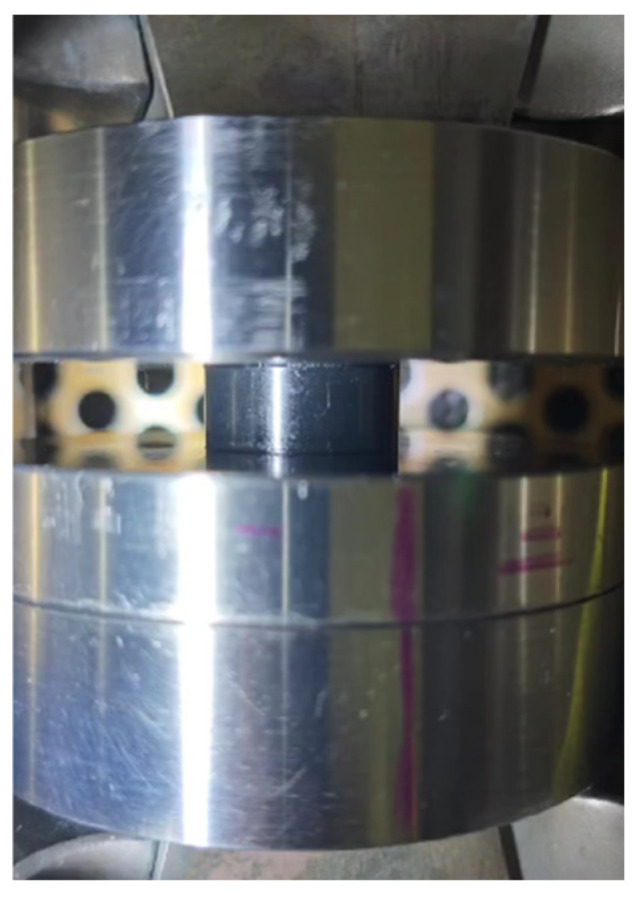
Compression device.

To evaluate temperature’s influence on the hyperelastic response of EPDM rubber after accelerated aging, uniaxial compression tests were carried out using a material testing machine with a temperature chamber, as shown in Figure 3. After accelerated aging, the EPDM specimens exhibited an increase in diameter, a reduction in height, uneven deformation, slightly irregular end surfaces, and minor circumferential bulging. Therefore, the dimensions of the aged specimens were remeasured, and the results are presented in Appendix A. The test temperatures in the chamber were set to 273 K, 283 K, 293 K, and 313 K.

A silicone lubricant was uniformly applied to the top and bottom surfaces of the EPDM specimens to minimize the friction between the rubber and compression plates during testing. In order to gain stable mechanical behavior and reduce the Mullins effect, EPDM specimens were subjected to four cycles of loading and unloading. A one-minute interval was set for deformation recovery between each cycle. All compression tests were conducted at a loading velocity of 10 mm/min. For each temperature condition, three specimens were tested for aged and unaged EPDM to assess data repeatability. The test results are provided in Appendix B. The average load–displacement data from the final cycle of the three specimens were used to represent the compression behavior of the EPDM rubber specimens. The nominal stress and strain were calculated based on the initial cross-sectional area and specimen height.

## 3. Results and Discussion

### 3.1. Test Results of Accelerated Aged EPDM Rubber at Different Temperatures

**Figure 4:**
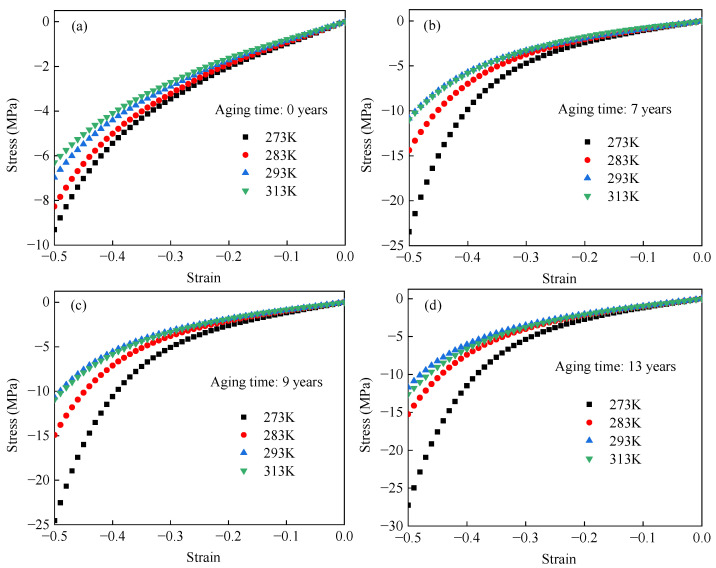
Uniaxial compressive stress–strain curves of EPDM rubber equivalent aged for (**a**) 0, (**b**) 7, (**c**) 9, and (**d**) 13 years at different temperatures.

**Figure 5:**
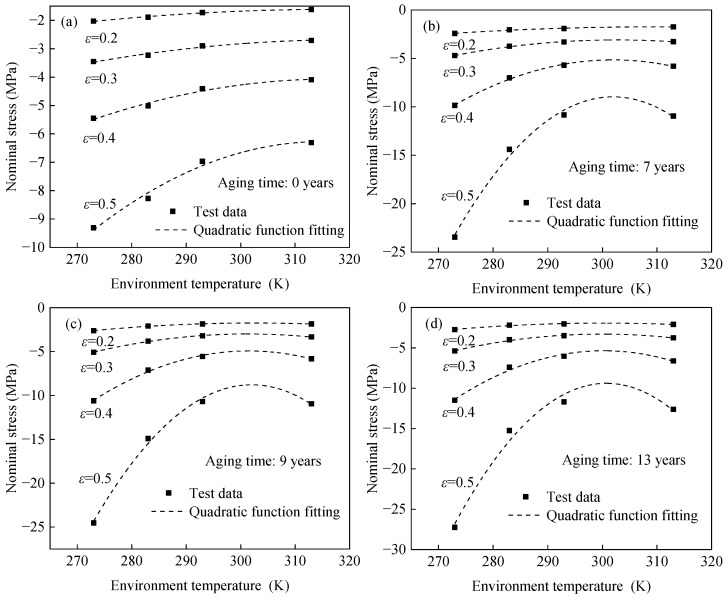
Stress–temperature curves of EPDM rubber equivalent aged (**a**) 0, (**b**) 7, (**c**) 9, and (**d**) 13 years at different strain values.

Figure 4 presents the nominal stress–strain responses of EPDM rubber at 273 K, 283 K, 293 K, and 313 K, corresponding to equivalent aging times of 0, 7, 9, and 13 years, respectively. In Figure 4a, the stiffness of unaged EPDM rubber decreases monotonically with increasing temperature. In contrast, Figure 4b–d show a more complex temperature dependence for aged EPDM rubber, where the stiffness initially decreases with increasing temperature and then increases. To more clearly show the change in EPDM rubber stiffness with temperature, Figure 5 presents nominal stress–temperature curves at strain levels of 0.2, 0.3, 0.4, and 0.5. From this figure, it can be clearly observed that the stiffness of unaged rubber decreases with increasing temperature, whereas the stiffness of aged rubber initially decreases and then increases as the temperature rises. Such results are consistent with the temperature-dependent hyperelastic behavior of carbon black-filled rubber reported in the literature [24,25,26,31].

The observed phenomenon can be attributed to the combined effects of the positive entropic elasticity of the rubber matrix and the negative enthalpic elasticity of the carbon black fillers [28,30,31]. Specifically, at lower temperatures, the enthalpic elasticity of EPDM rubber dominates. As the temperature increases, the contribution of enthalpic elasticity decreases, while the contribution of entropic elasticity increases. The interplay between these two mechanisms results in the non-monotonic temperature dependence of stiffness, which first decreases and then increases with temperature. Compared to unaged EPDM rubber, this transition appears earlier in aged samples. This is because thermal aging disrupts the filler–rubber molecular network and the filler–filler network, thereby reducing the enthalpic energy contribution associated with the carbon black fillers and consequently allowing the entropic elasticity to dominate more rapidly.

### 3.2. Test Results of Different Accelerated Aged EPDM Rubber Specimens at Constant Temperatures

**Figure 6:**
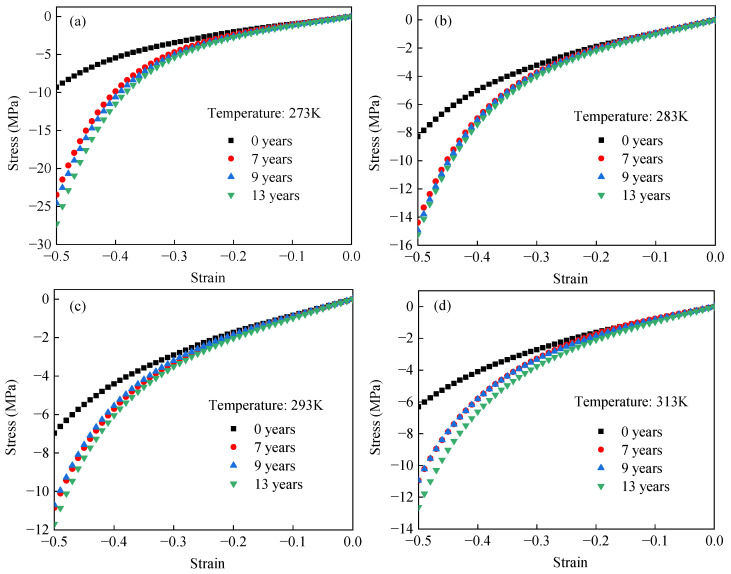
Uniaxial compressive stress–strain curves of EPDM rubber with different equivalent aging times at (**a**) 273 K, (**b**) 283 K, (**c**) 293 K, and (**d**) 313 K.

**Figure 7:**
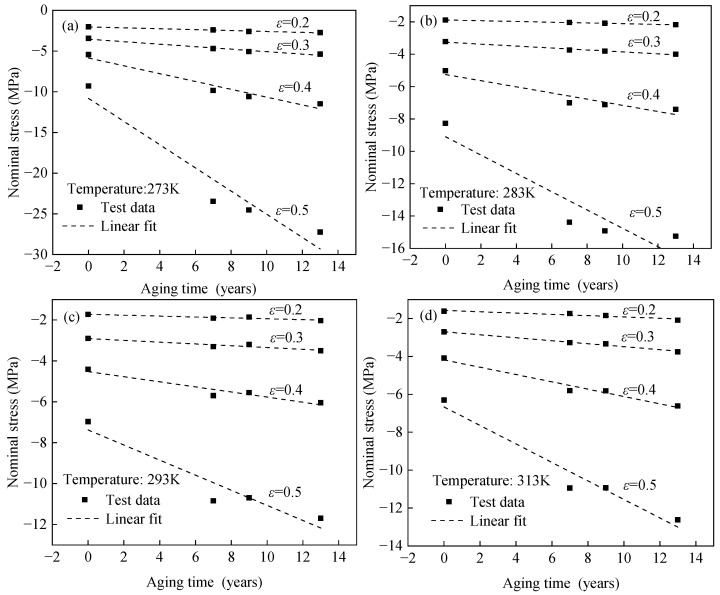
Stress equivalent aging times curves of EPDM rubber with different strain values at (**a**) 273 K, (**b**) 283 K, (**c**) 293 K, and (**d**) 313 K.

The hyperelastic behavior of unaged and aged EPDM rubber at constant temperatures was compared, as shown in Figure 6. Figure 6 shows that the stiffness of EPDM rubber increases monotonically with aging time under different temperatures. To clearly characterize this relationship, Figure 7 presents the stress–aging time relationship of EPDM rubber at constant temperatures and given strain levels of 0.2, 0.3, 0.4, and 0.5. Figure 7 indicates that the stiffness of EPDM rubber increases approximately linearly with aging time under different temperatures. According to the relationship between the molecular network structure of rubber and its stiffness during aging, crosslinking tends to increase stiffness, whereas chain scission leads to a decrease in stiffness [41,53,54]. Within the studied aging duration, crosslinking is considered the dominant mechanism in the aging process of EPDM rubber, which explains the observed monotonic increase in stiffness with aging time.

### 3.3. Discussion on Relationship Between Accelerated Aging and Service Temperature

**Figure 8:**
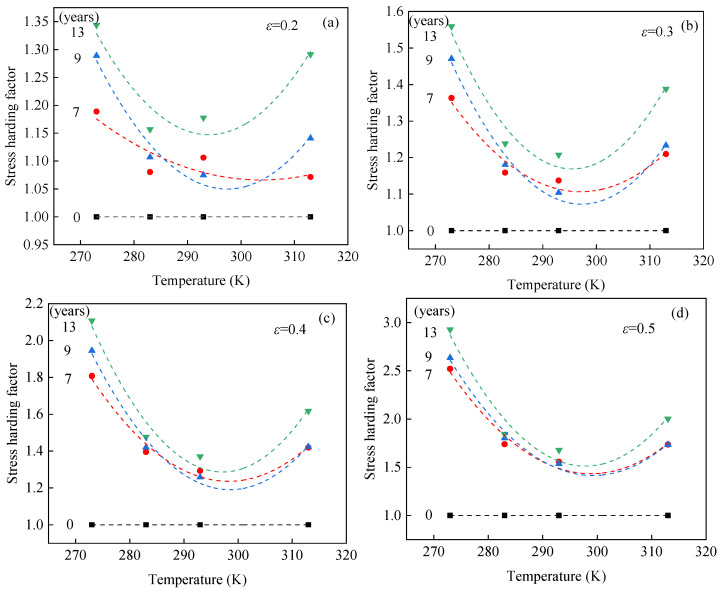
Stress hardening factor–temperature curves of EPDM rubber with different equivalent aging times at strain values of (**a**) 0.2, (**b**) 0.3, (**c**) 0.4, and (**d**) 0.5.

In order to analyze the effects of temperature on aging behavior, the concept of a stress hardening factor was introduced. This factor characterizes the stress hardening behavior induced by aging at a given strain, and its expression is given as follows:

(2) $$\delta =\sigma (\varepsilon_{g},t_{g})/\sigma_{0}(\varepsilon_{g},t_{0})$$

where $\sigma (\varepsilon_{g},t_{g})$ represents the nominal stress of EPDM rubber at a given strain $\varepsilon_{g}$, and the given aging time $t_{g}$. $\sigma_{0}(\varepsilon_{g},t_{0})$ denotes the nominal stress of unaged EPDM rubber at the given strain $\varepsilon_{g}$ and given aging time $t_{0}$. A similar definition has been used to characterize the temperature dependence of filler effects in filled rubbers [31] and the influence of temperature on irradiation effects in silicone rubber foams [33]. Figure 8 shows the curves of the stress hardening factor versus temperature at given strain values of 0.2, 0.3, 0.4, and 0.5. It shows the stress hardening factor of aged EPDM rubber initially decreases and then increases with temperature. This result indicates that the stiffness of aged EPDM rubber first decreases and then increases with increasing temperature, which is consistent with the analysis presented in Section 3.1. In addition, it is observed that the variation in the stress hardening factor with temperature becomes more pronounced with increasing aging time. This indicates that the stiffness of EPDM rubber increases with aging time, which is consistent with the findings presented in Section 3.2.

## 4. Constitutive Modeling

### 4.1. Hyperelastic Constitutive Relation

Hyperelastic materials usually have nonlinear elastic behaviors under large deformation. The constitutive behavior is determined by the strain energy function. The strain energy function is expressed as a function of the deformation gradient tensor ***F***, denoted as *W*(***F***). The strain energy function can also be represented as a function of the right stretch tensor ***U***, i.e., *W*(***U***). For large deformation, the right Cauchy–Green deformation tensor ***C*** = ***U***^2^ = ***F***ᵀ***F*** is commonly used to describe material deformation. Rubber is a typically isotropic and homogeneous hyperelastic material. The strain energy function can be further simplified as a function of the three invariants *I*_1_, *I*_2_, and *I*_3_ of the right Cauchy–Green tensor, i.e., *W*(*I*_1_, *I*_2_, *I*_3_). The three invariants are as follows:

(3) $$\left\{\begin{array}{l} I_{1}=\mathrm{tr}C=\lambda_{1}^{2}+\lambda_{2}^{2}+\lambda_{3}^{2}\\ I_{2}=\frac{1}{2}[{\left(\mathrm{tr}C\right)}^{2}-(\mathrm{tr}C^{2})]=\lambda_{1}^{2}\lambda_{2}^{2}+\lambda_{2}^{2}\lambda_{3}^{2}+\lambda_{1}^{2}\lambda_{3}^{2}\\ I_{3}=\det C=\lambda_{1}^{2}\lambda_{2}^{2}\lambda_{3}^{2} \end{array}\right.$$

where *λ*_1_, *λ*_2_, and *λ*_3_ are the three eigenvalues of the stretch tensor ***U***, which are physically interpreted as the three principal stretch ratios of the material.

Rubber materials are typically incompressible, *I*_3_ = 1, and the principal stress $\sigma_{i}$ (*i* = 1, 2, 3) can be expressed as follows:

(4) $$\sigma_{i}=\frac{\partial W}{\partial \lambda_{i}}-p\lambda_{i}^{-1}=\frac{\partial W}{\partial I_{1}}\frac{\partial I_{1}}{\partial \lambda_{i}}+\frac{\partial W}{\partial I_{2}}\frac{\partial I_{2}}{\partial \lambda_{i}}-p\lambda_{i}^{-1}$$

where $\sigma_{i}$ (*i* = 1, 2, 3) denotes the nominal stress in the principal directions, and *p* is the hydrostatic pressure associated with the incompressibility constraint.

For uniaxial compression, assuming the stress conditions *σ*_2_ = *σ*_3_ = 0 and the deformation characteristic *λ*_1_ = *λ*, *λ*_2_ = *λ*_3_ = *λ*^−1/2^, substituting into Equation (4) gives the corresponding constitutive relation:

(5) $$\sigma_{1}=2\left(\frac{\partial W}{\partial I_{1}}-\frac{\partial W}{\partial I_{2}}\cdot \lambda^{-1}\right)(\lambda -\lambda^{-2})$$

### 4.2. Hyperelastic Constitutive Model for EPDM Rubber

#### 4.2.1. Model Evaluation Method

In order to characterize the hyperelastic behavior of rubber at different aging times and temperatures, an appropriate hyperelastic model should be established. A hyperelastic model, which can accurately fit the hyperelastic behavior of EPDM rubber under various aging times and temperatures, should be proposed. In this paper, the hyperelastic model parameters were obtained by fitting the experimental data using the nonlinear least squares method. The coefficient of determination R^2^ was used to evaluate model performance. R^2^ is calculated by the residual sum of squares (RSS) and the total sum of squares (TSS). The specific calculation formula is given by the following:

(6) $$\left\{\begin{array}{l} R^{2}=1-\mathrm{RSS}/\mathrm{TSS}\\ \mathrm{TSS}=\sum_{i=1}^{n}\left(\sigma_{\mathrm{test}}-{\bar{\sigma }}_{\mathrm{test}}\right)\\ \mathrm{RSS}=\sum_{i=1}^{n}\left(\sigma_{\mathrm{fit}}-\sigma_{\mathrm{test}}\right) \end{array}\right.$$

where $\sigma_{\mathrm{test}}$ is the experimental value, ${\bar{\sigma }}_{\mathrm{test}}$ is the average of the experimental values, $\sigma_{\mathrm{fit}}$ is the model prediction value, and *n* is the number of experimental data points. The closer R^2^ is to 1, the better the model fits experimental data. The nonlinear least squares method determines the optimal fit by minimizing the residual sum of squares (RSS). For EPDM rubber, the absolute error of the compressive stress–strain curve is small for strains below 0.3. This means that there is a small effect on the RSS. As a result, the fitting process may compromise the relative error of stress at a strain below 0.3. Considering practical engineering applications, a relative error within 20% is generally acceptable. The relative error of the model is also analyzed to evaluate the hyperelastic constitutive relationship. The rubber surface becomes uneven after pre-aging, leading to highly unstable test errors during the initial stage of experimentation. Therefore, relative errors at strains below 0.1 are excluded from the subsequent analysis.

#### 4.2.2. Evaluation of Rivlin Models of Different Orders

Hyperelastic constitutive models are generally classified into molecular chain network models and phenomenological models. Because phenomenological models are simpler than molecular chain network models, a phenomenological model is adopted to characterize the hyperelastic behavior of EPDM rubber. The most representative phenomenological constitutive model is the polynomial model proposed by Rivlin, whose strain energy function is expressed as follows:

(7) $$W=\sum_{i,j=0}^{N}C_{\mathrm{ij}}{\left(I_{1}-3\right)}^{i}{\left(I_{2}-3\right)}^{j}$$

where *C_ij_* denotes the material constants.

Taking the Rivlin term with *i* = 1 and *j* = 0, the model reduces to the Neo–Hookean model [15]. It is the simplest form with only one parameter, and the constitutive equation under uniaxial loading is expressed as follows:

(8) $$\sigma_{1}=2\cdot C_{10}(\lambda -\lambda^{-2})$$

**Figure 9:**
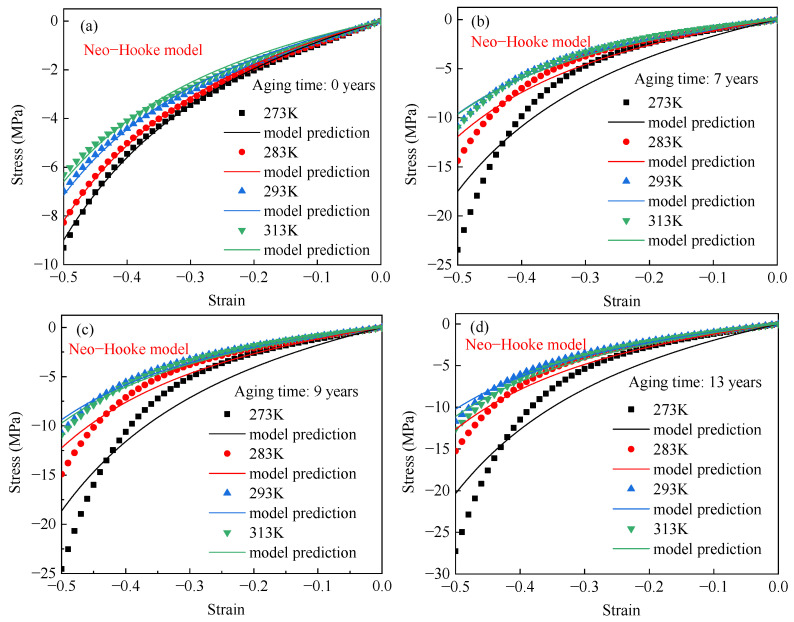
The stress–strain data of EPDM rubber aged for (**a**) 0 years, (**b**) 7 years, (**c**) 9 years, and (**d**) 13 years at different temperatures and the fitting curves of the Neo–Hooke model.

The Neo–Hookean model was used to fit the compressive stress–strain curves of EPDM rubber aged for different times. The fitting results are shown in Figure 9. It shows that the Neo–Hookean model can accurately describe the relationship between compressive stress and strain for unaged EPDM rubber. In contrast, significant deviations from the experimental results are observed for aged EPDM rubber. For unaged EPDM rubber, the model provides an R^2^ of 99.18% and a maximum relative error of 19.66%, as shown in Table 2. For EPDM rubber aged for 7, 9, and 13 years, the R^2^ values are 91.33%, 92.02%, and 91.02%, respectively. Meanwhile, the maximum relative errors reach 60.14%, 56.23%, and 65.04%, respectively. This indicates that the Neo–Hookean model is suitable for describing the temperature-dependent behavior of unaged EPDM rubber, but it is not applicable to aged EPDM rubber.

Setting Rivlin terms with *i* = 1, *j* = 0 and *i* = 0, *j* = 1, the Mooney–Rivlin model is obtained [12]. As the model has two parameters, it has improved fitting accuracy compared to the Neo–Hookean model. The uniaxial loading constitutive equation is expressed as follows:

(9) $$\sigma_{1}=2\cdot C_{10}(\lambda -\lambda^{-2})+2\cdot C_{01}(1-\lambda^{-3})$$

**Figure 10:**
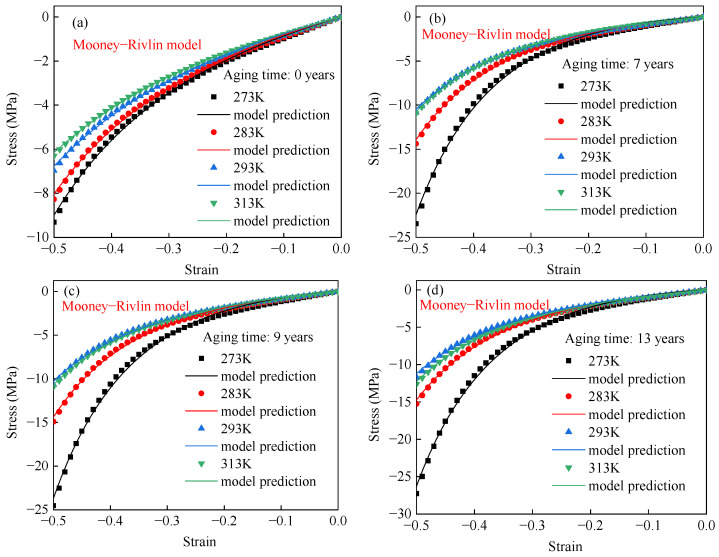
The stress–strain curves of EPDM rubber aged for (**a**) 0 years, (**b**) 7 years, (**c**) 9 years, and (**d**) 13 years at different temperatures using the Mooney–Rivlin model.

Figure 10 shows the Mooney–Rivlin model fitting results of the compressive stress–strain curves of EPDM rubber aged for different times. This shows that the Mooney–Rivlin model can accurately capture the compressive behavior of EPDM rubber aged for different times under varying conditions. According to Table 2, the R^2^ value of the Neo–Hookean model for EPDM rubber is 99.60%, and the maximum relative errors for the compression data of EPDM rubber aged for 7 years, 9 years, and 13 years are 38.41%, 36.20%, and 38.98%, respectively. These errors are mainly distributed in the small deformation range. Though the Mooney–Rivlin model provides an accurate description of large deformations for EPDM rubber, it remains insufficient in accurately describing small-strain behavior.

In order to improve the Mooney–Rivlin model’s adequacy in describing the small deformation behavior of EPDM rubber, Rivlin terms corresponding to *i* = 2, *j* = 0 are added to the model, which results in an improved Mooney–Rivlin model. The constitutive expression under uniaxial loading is given by the following:

(10) $$\sigma_{1}=2(\lambda -\lambda^{-2})(C_{10}+2C_{20}(\lambda^{2}+2\lambda^{-1}-3)+C_{01}\cdot \lambda^{-1})$$

**Figure 11:**
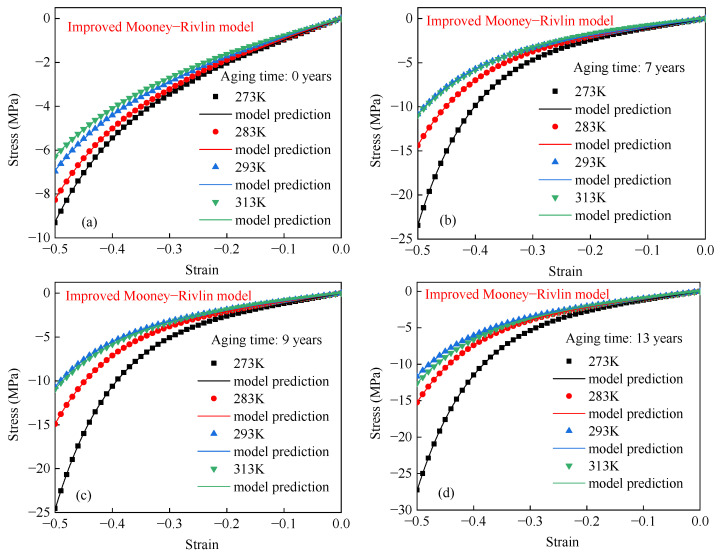
The stress–strain curves of EPDM rubber aged for (**a**) 0 years, (**b**) 7 years, (**c**) 9 years, and (**d**) 13 years at different temperatures using the improved Mooney–Rivlin model.

The improved Mooney–Rivlin model was used to fit the compression data of EPDM rubber under different experimental conditions. The fitting results are shown in Figure 11. Figure 11 shows that the improved Mooney–Rivlin model can effectively describe the compressive behavior of EPDM rubber. Table 2 shows that the R^2^ values of the improved Mooney–Rivlin model under all experimental conditions exceed 99.99%, and the maximum relative error remains less than 6%. This indicates that the improved Mooney–Rivlin model is effective in describing the hyperelastic behavior of EPDM rubber aged for different times and temperatures.

### 4.3. Effects of Temperature and Accelerated Aging on Model Parameters

The improved Mooney–Rivlin model can accurately describe the temperature-dependent hyperelastic behavior of both unaged and aged EPDM rubber. The corresponding material parameters *C*_10_, *C*_01_, and *C*_20_ at different temperatures and under different aging time conditions are shown in Table 3. However, the improved Mooney–Rivlin model does not provide a unified explicit expression for describing the variation in parameters *C*_10_, *C*_01_, and *C*_20_. Therefore, it is necessary to establish a mathematical function of temperature and aging time to explicitly characterize the parameters *C*_10_, *C*_01_, and *C*_20_ of the improved Mooney–Rivlin model.

According to the analysis in Section 3, three significant phenomena can be observed: (1) The stiffness of EPDM rubber increases approximately linearly with aging time. (2) The stiffness of EPDM rubber exhibits an approximately quadratic dependence on temperature. (3) The effects of temperature on aging behavior approximately follow a quadratic trend. Since the modified Mooney–Rivlin model parameters *C*_10_, *C*_01_, and *C*_20_ are related to the stiffness of rubber, it is assumed that these parameters follow the following relationships:

(11) $$\left\{\begin{array}{l} C_{10}(T,t)=k_{1}(T)t+b_{1}(T)\\ C_{20}(T,t)=k_{2}(T)t+b_{2}(T)\\ C_{01}(T,t)=k_{3}(T)t+b_{3}(T) \end{array}\right.$$

If the parameter *t* = 0, then $C_{10}(T,0)=b_{1}(T)$, $C_{20}(T,0)=b_{2}(T)$, and $C_{01}(T,0)=b_{3}(T)$ according to Equation (11). $k_{i}(T)$ and $b_{i}(T)$ are related to aged and unaged rubber, respectively, and their formulations are as follows:

(12) $$\left\{\begin{array}{l} k_{i}(T)=a_{1i}T^{2}+b_{1i}T+c_{1i}\\ b_{i}(T)=a_{0i}T^{2}+b_{0i}T+c_{0i} \end{array}\right.\;\;i=1,2,3$$

where $a_{1i}$, $b_{1i}$, and $c_{1i}$ (*i* = 1, 2, 3) represent the temperature coefficients associated with aged rubber, and $a_{0i}$, $b_{0i}$, and $c_{0i}$ (*i* = 1, 2, 3) denote the temperature coefficients corresponding to unaged rubber.

**Figure 12:**
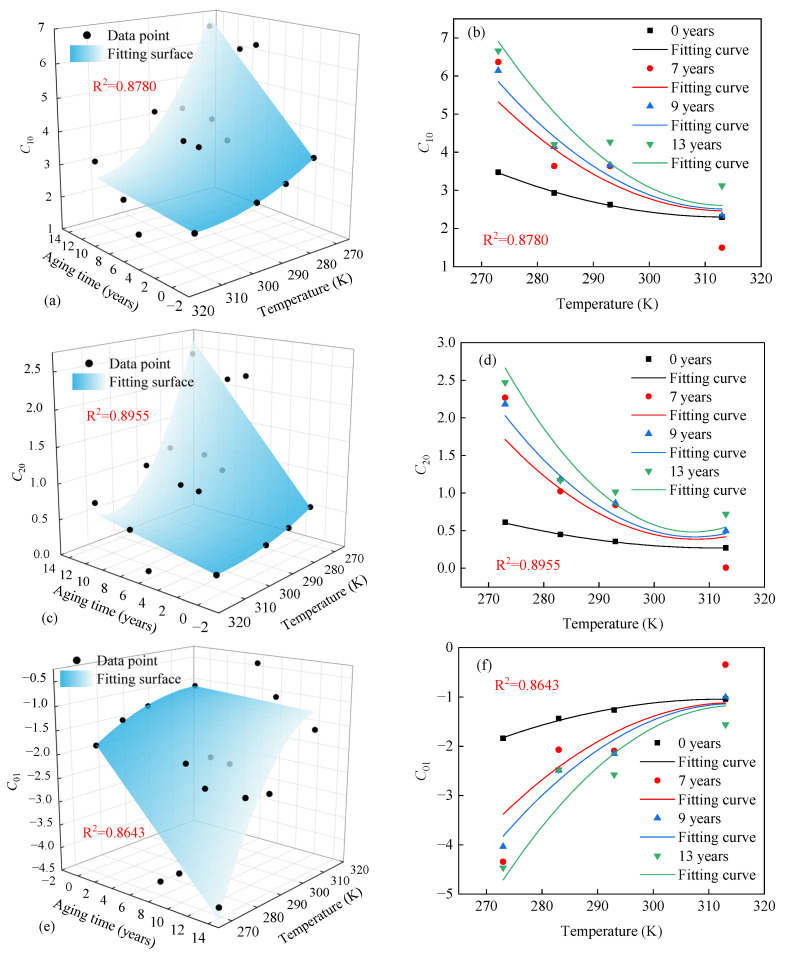
Variations in parameters *C*_10_, *C*_20_, and *C*_01_ with different temperatures and aging times are expressed in (**a**–**c**) three dimensions and (**d**–**f**) two dimensions.

Equation (11) was fitted with the experimental data using the nonlinear least squares method, and the fitting results are shown in Figure 12a–c. The fitted values of *C*_10_, *C*_01_, and *C*_20_ are presented in a two-dimensional graph, as shown in Figure 12d–f. In Figure 12, although the fitting results deviate from several individual data points, the whole trend is consistent with the distribution of the data. The fitted surface R^2^ values for *C*_10_, *C*_01_, and *C*_20_ are 0.878, 0.8955, and 0.8643, respectively. This shows that Equation (11) describes the temperature and aging-dependent evolution of these parameters well. The specific values of the fitted model parameters are listed in Table 4. By substituting the parameters into Equation (11), the temperature-dependent hyperelastic constitutive model for aged rubber can be expressed by Equations (10) and (11).

### 4.4. Model Verification

**Figure 13:**
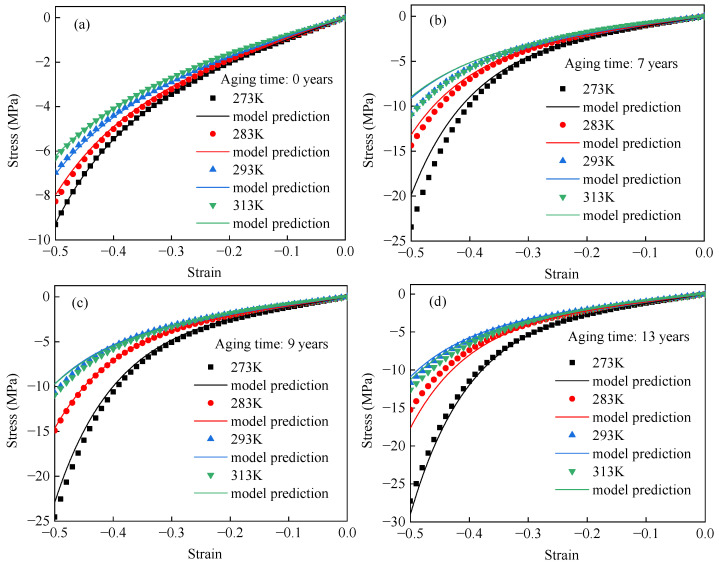
A comparison between the prediction results of the proposed model and the stress–strain test data of EPDM aged for (**a**) 0a, (**b**) 7a, (**c**) 9a, and (**d**) 13a at different temperatures.

Figure 13 shows a comparison of the experimental and predicted curves. It indicates that the predicted curves are consistent with the experimental data. The maximum relative error between the model results and experimental data is presented in Table 5. The maximum relative errors of the proposed model predicting the compressive behavior of EPDM aged for 0, 7, 9, and 13 years are 3.73%, 18.25%, 11.02%, and 15.71%, respectively. This indicates that the model can predict the temperature-dependent compressive stress–strain curves of unaged and aged EPDM.

## 5. Conclusions

An accelerated aging test considering the time–temperature equivalence principle was conducted to perform EPDM equivalent aging for 7, 9, and 13 years. The effects of temperature on the hyperelastic mechanical behavior of EPDM rubber after accelerated aging were studied. The conclusions are as follows:

(1) The experimental data shows that the stiffness of EPDM rubber aged for different times initially decreases and then increases with increasing temperature. The critical transition temperature decreases with increasing aging time. In addition, the stiffness of EPDM rubber increases approximately linearly with aging time at different temperatures.
(2) The stress hardening factor is defined to characterize the effects of temperature on the aging effects of EPDM rubber. The results indicate that the stress hardening factor initially decreases and then increases with testing temperature.
(3) The temperature-dependent hyperelastic behavior of aged rubber is analyzed using the Neo–Hooke model, the Mooney–Rivlin model, and an improved Mooney–Rivlin model. The results show that the improved Mooney–Rivlin model can accurately describe temperature-dependent hyperelastic behavior.
(4) The parameters of the improved Mooney–Rivlin model were extended to incorporate the effects of temperature and aging time. Moreover, the experimental data were used to validate the proposed model, and the results show that the model can accurately capture the compression response of both unaged and aged EPDM rubber, particularly within the compression strain range of 50%.

## Figures and Tables

**Table 1:** Formulation of the EPDM compounds.

Ingredients	Value [phr]
EPDM EPT4045	100
Carbon black N330	30
Organo-modified montmorillonite	25
Compatibilizer EPDM-g-MAH	10
Zinc oxide	5
Antioxidant poly(1,2-dihydro-2,2,4-trimethyl-quinoline)	3
Vulcanizator 2,5-Dimethyl-2,5-di(tert-butylperoxy) hexane	3

**Table 2:** The fitting determination coefficients and maximum relative error values of three hyperelastic constitutive models.

Aging Time (Years)	Temperature (K)	Neo–Hooke Model		Mooney–Rivlin Model		Improved Mooney–Rivlin Model	
		R^2^ (%)	The Maximum Relative Error (%)	R^2^ (%)	The Maximum Relative Error (%)	R^2^ (%)	The Maximum Relative Error (%)
0	273	99.83	12.90	99.83	12.99	99.99	2.08
	283	99.82	14.98	99.88	11.33	99.99	2.13
	293	99.44	19.25	99.90	10.11	99.99	2.06
	313	99.18	19.66	99.93	8.16	99.99	1.67
7	273	91.33	60.14	99.60	38.41	1.00	5.44
	283	96.36	27.56	99.80	22.43	1.00	0.87
	293	98.64	11.63	99.76	19.24	1.00	1.08
	313	98.26	24.68	99.97	8.16	1.00	2.42
9	273	92.02	56.23	99.68	36.20	1.00	2.06
	283	96.06	27.80	99.75	23.55	1.00	2.19
	293	98.48	12.44	99.74	19.64	1.00	1.91
	313	98.59	16.00	99.92	10.97	1.00	2.58
13	273	91.02	65.04	99.66	38.98	1.00	3.52
	283	96.47	26.45	99.77	23.41	1.00	0.97
	293	98.47	12.54	99.70	21.00	1.00	1.15
	313	98.32	15.85	99.88	15.10	1.00	1.31

**Table 3:** The fitting parameters of the improved Mooney–Rivlin model according to the test data of EPDM rubber at different temperatures and aging times.

Aging Time (Years)	Temperature (K)	*C*_10_	*C*_20_	*C*_01_
0	273	3.475	0.610	−1.838
	283	2.930	0.449	−1.439
	293	2.625	0.355	−1.262
	313	2.297	0.270	−1.038
7	273	6.366	2.268	−4.344
	283	3.638	1.023	−2.070
	293	3.636	0.841	−2.095
	313	1.496	0.301	−0.345
9	273	6.144	2.181	−4.038
	283	4.144	1.183	−2.487
	293	3.661	0.864	−2.150
	313	2.329	0.497	−1.006
13	273	6.661	2.471	−4.464
	283	4.205	1.168	−2.472
	293	4.272	1.016	−2.574
	313	3.124	0.719	−1.560

**Table 4:** The characterization parameters related to the temperature and aging time of the improved Mooney–Rivlin model.

Aging-Related		Unaged-Related	
Parameter	Value	Parameter	Value
*a*_11_	1.4797 × 10^−4^	*a*_01_	7.0118 × 10^−4^
*b*_11_	−0.0928	*b*_01_	−0.4399
*c*_11_	14.5606	*c*_01_	71.3091
*a*_12_	1.2734 × 10^−4^	*a*_02_	2.2128 × 10^−4^
*b*_12_	−0.0781	*b*_02_	−0.1380
*c*_12_	11.9758	*c*_02_	21.7822
*a*_13_	−1.1580 × 10^−4^	*a*_03_	−5.0176 × 10^−4^
*b*_13_	0.07315	*b*_03_	0.3135
*c*_13_	−11.5622	*c*_03_	−50.0245

**Table 5:** The maximum relative errors between the prediction results of the proposed model and the experimental data.

Aging Time (Years)	Temperature (K)	The Maximum Relative Error (%)
0	273	2.38
	283	3.73
	293	3.00
	313	1.48
7	273	15.21
	283	8.43
	293	15.92
	313	18.25
9	273	7.00
	283	4.90
	293	9.20
	313	11.02
13	273	6.43
	283	15.71
	293	6.80
	313	10.58

**Figure A1:**
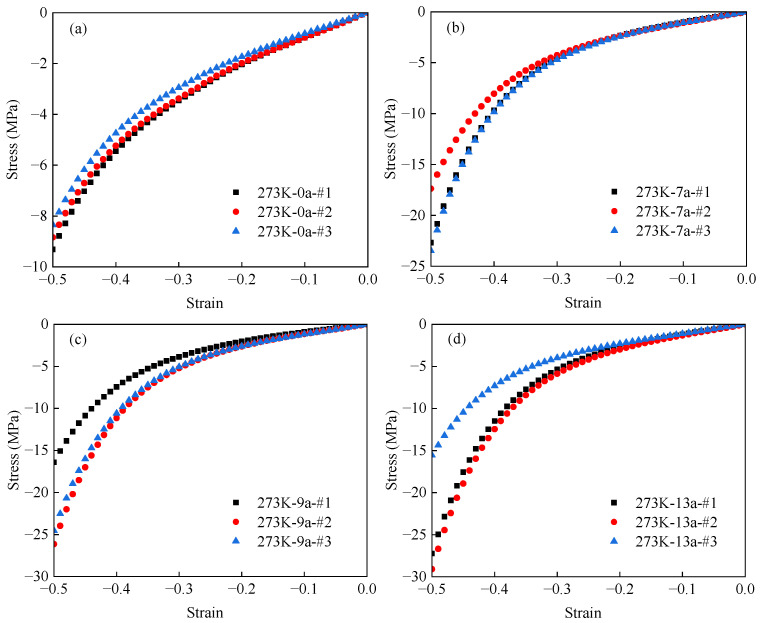
Repeatability of compression test results at 273 K for EPDM rubber after equivalent aging periods of (**a**) 0, (**b**) 7, (**c**) 9, and (**d**) 13 years.

**Figure A2:**
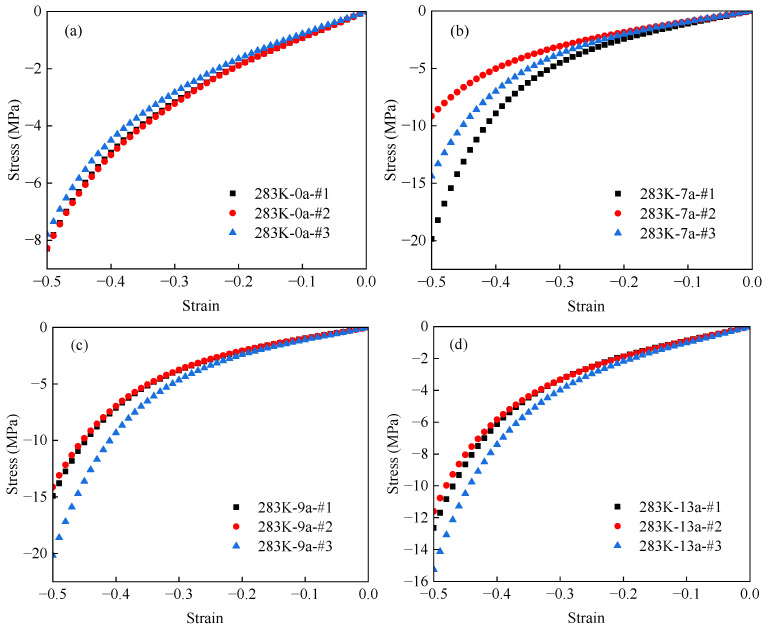
Repeatability of compression test results at 283 K for EPDM rubber after equivalent aging periods of (**a**) 0, (**b**) 7, (**c**) 9, and (**d**) 13 years.

**Figure A3:**
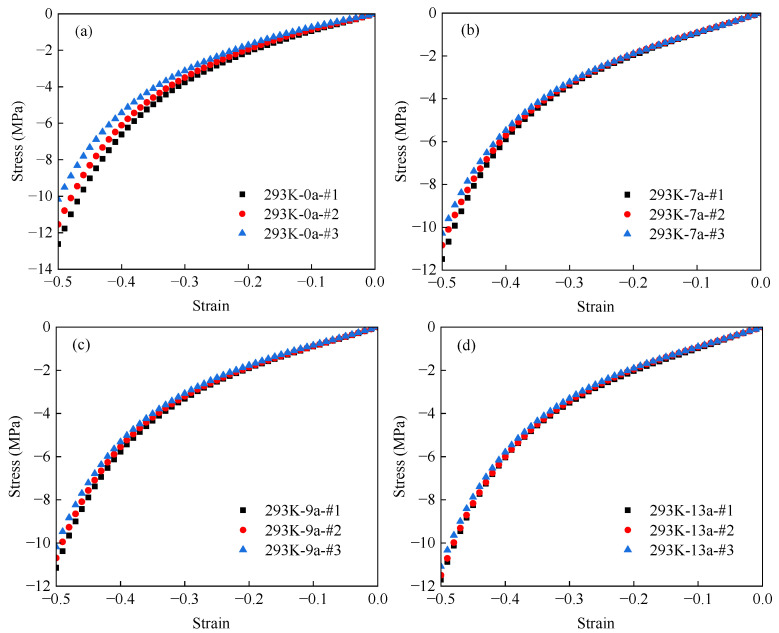
Repeatability of compression test results at 293 K for EPDM rubber after equivalent aging periods of (**a**) 0, (**b**) 7, (**c**) 9, and (**d**) 13 years.

**Figure A4:**
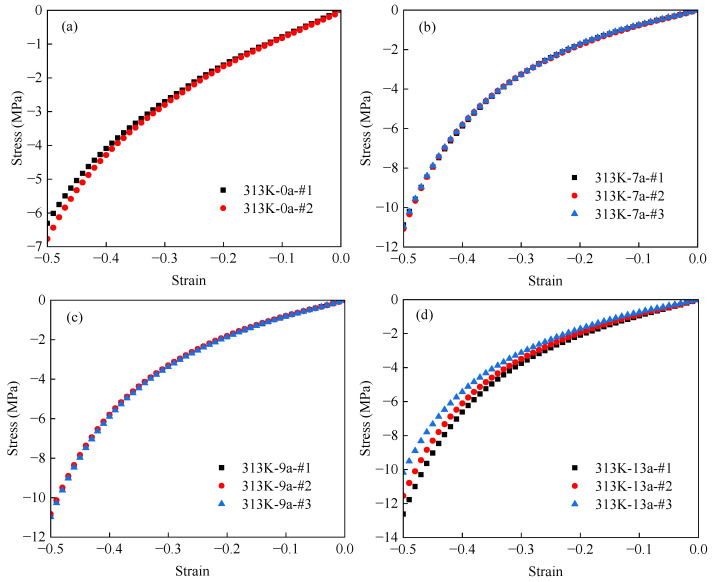
Repeatability of compression test results at 313 K for EPDM rubber after equivalent aging periods of (**a**) 0, (**b**) 7, (**c**) 9, and (**d**) 13 years.

## Data Availability

The original contributions presented in this study are included in the article; further inquiries can be directed to the corresponding author.

## Appendix A

In this appendix, the specific dimensions of the unaged and aged EPDM specimens are provided, as shown in Table A1, Table A2, Table A3 and Table A4.

**Table A1:** Detailed dimensions of unaged EPDM specimens.

Temperature (K)	Specimen Number	Diameter (mm)	Height (mm)
273 K	273K-0a-#1	28.48	12.35
	273K-0a-#2	28.63	12.13
	273K-0a-#2	28.81	12.32
283 K	283K-0a-#1	28.66	12.31
	283K-0a-#2	28.67	12.14
	283K-0a-#3	28.84	12.30
293 K	293K-0a-#1	29.02	12.27
	293K-0a-#2	29.00	12.25
	293K-0a-#3	28.76	12.17
313 K	313K-0a-#1	28.85	12.30
	313K-0a-#2	28.54	12.13

**Table A2:** Detailed dimensions of EPDM specimens aged equivalently for 7 years.

Temperature (K)	Specimen Number	Diameter (mm)	Height (mm)
273 K	273K-7a-#1	30.53	10.90
	273K-7a-#2	30.03	11.61
	273K-7a-#2	30.45	11.43
283 K	283K-7a-#1	30.44	11.03
	283K-7a-#2	30.41	11.05
	283K-7a-#3	30.59	10.92
293 K	293K-7a-#1	30.27	11.03
	293K-7a-#2	29.61	11.56
	293K-7a-#3	30.38	10.93
313 K	313K-7a-#1	30.49	11.20
	313K-7a-#2	30.59	10.88
	313K-7a-#3	30.57	11.01

**Table A3:** Detailed dimensions of EPDM specimens aged equivalently for 9 years.

Temperature (K)	Specimen Number	Diameter (mm)	Height (mm)
273 K	273K-9a-#1	30.38	11.00
	273K-9a-#2	30.56	11.01
	273K-9a-#2	30.71	10.99
283 K	283K-9a-#1	30.45	11.06
	283K-9a-#2	30.88	10.91
	283K-9a-#3	31.00	11.03
293 K	293K-9a-#1	30.59	10.82
	293K-9a-#2	30.54	10.86
	293K-9a-#3	30.40	10.89
313 K	313K-9a-#1	30.67	10.91
	313K-9a-#2	30.65	10.85
	313K-9a-#3	30.72	11.25

**Table A4:** Detailed dimensions of EPDM specimens aged equivalently for 13 years.

Temperature (K)	Specimen Number	Diameter (mm)	Height (mm)
273 K	273K-13a-#1	31.03	10.80
	273K-13a-#2	30.66	11.04
	273K-13a-#2	29.83	11.49
283 K	283K-13a-#1	30.49	11.20
	283K-13a-#2	30.37	10.77
	283K-13a-#3	30.87	10.76
293 K	293K-13a-#1	30.50	10.71
	293K-13a-#2	30.82	10.78
	293K-13a-#3	30.75	11.01
313 K	313K-13a-#1	30.69	11.04
	313K-13a-#2	30.81	10.82
	313K-13a-#3	31.17	10.97

## Appendix B

In this appendix, the repeatability of the compression test results at different temperatures for EPDM rubber after equivalent aging periods of (a) 0, (b) 7, (c) 9, and (d) 13 years is provided, as shown in Figure A1, Figure A2, Figure A3 and Figure A4.

## References

[1] Hough P., van der Aar N., Qiu Z. (2020). Compounding and Mixing Methodology for Good Performance of EPDM in Tire Sidewalls. Tire Sci. Technol..

[2] Wang R., Zhang Y., Wang N., Wu Y. (2024). Rate-Dependent Tensile Properties of Aluminum-Hydroxide-Enhanced Ethylene Propylene Diene Monomer Coatings for Solid Rocket Motors. Materials.

[3] Guo M., Li J., Xi K., Liu Y., Ji J. (2019). Effect of Multi-Walled Carbon Nanotubes On Thermal Stability and Ablation Properties of EPDM Insulation Materials for Solid Rocket Motors. Acta Astronaut..

[4] Xie Z., Chen J., Zhang K. (2024). Lifetime Prediction of EPDM Sealing Materials with Thermal Aging Under Constant Compression. J. Phys. Conf. Ser..

[5] Huang X., Gu J., Li M., Yu X., Liu Y., Xu G. (2023). A Leakage Prediction Model for Sealing Performance Assessment of EPDM O-Rings under Irradiation Conditions. Polymers.

[6] Zhou Y., Qiu L., Xu Z., Huang S., Nie J., Yin H., Tu F., Zhao Z. (2024). Thermal Oxidative Aging and Service Life Prediction of Commercial Ethylene–Propylene–Diene Monomer Spacer Damping Composites for High-Voltage Transmission Lines. Polymers.

[7] Hu Q., Chen Q., Song P., Gong X., Chen J., Zhao Y. (2023). Performance of Thermal-Oxidative Aging on the Structure and Properties of Ethylene Propylene Diene Monomer (EPDM) Vulcanizates. Polymers.

[8] Jost C., Lacuve M., Haller S., Espuche E., Colin X. (2025). Influence of Thermo-Oxidative Aging On the Structure and the Water Transport Properties of Sulfur-Crosslinked EPDM. Polym. Degrad. Stab..

[9] Assink R.A., Gillen K.T., Sanderson B. (2002). Monitoring the Degradation of a Thermally Aged EPDM Terpolymer by 1 H NMR Relaxation Measurements of Solvent Swelled Samples. Polymer.

[10] Yeoh O.H. (1990). Characterization of Elastic Properties of Carbon-Black-Filled Rubber Vulcanizates. Rubber Chem. Technol..

[11] Ogden R.W. (1972). Large Deformation Isotropic Elasticity: On the Correlation of Theory and Experiment for Compressible Rubberlike Solids. Proc. R. Soc. A.

[12] Mooney M. (1940). A Theory of Large Elastic Deformation. J. Appl. Phys..

[13] Arruda E.M., Boyce M.C. (1993). A Three-Dimensional Constitutive Model for the Large Stretch Behavior of Rubber Elastic Materials. J. Mech. Phys. Solids.

[14] Miehe C. (2004). A Micro-Macro Approach to Rubber-Like Materials–Part I: The Non-Affine Micro-Sphere Model of Rubber Elasticity. J. Mech. Phys. Solids.

[15] Treloar L.R.G. (1943). The Elasticity of a Network of Long-Chain Molecules-II. Trans. Faraday Soc..

[16] He H., Zhang Q., Zhang Y., Chen J., Zhang L., Li F. (2022). A Comparative Study of 85 Hyperelastic Constitutive Models for Both Unfilled Rubber and Highly Filled Rubber Nanocomposite Material. Nano Mater. Sci..

[17] Dal H., Açıkgöz K., Badienia Y. (2021). On the Performance of Isotropic Hyperelastic Constitutive Models for Rubber-Like Materials: A State of the Art Review. Appl. Mech. Rev..

[18] Beda T. (2014). An Approach for Hyperelastic Model-Building and Parameters Estimation a Review of Constitutive Models. Eur. Polym. J..

[19] Steinmann P., Hossain M., Possart G. (2012). Hyperelastic Models for Rubber-Like Materials: Consistent Tangent Operators and Suitability for Treloar’s Data. Arch. Appl. Mech..

[20] Lion A. (1997). On the Large Deformation Behaviour of Reinforced Rubber at Different Temperatures. J. Mech. Phys. Solids.

[21] Holzapfel G.A., Simo J.C. (1996). Entropy Elasticity of Isotropic Rubber-Like Solids at Finite Strains. Comput. Methods Appl. Mech. Eng..

[22] Lu S.C.H., Pister K.S. (1975). Decomposition of Deformation and Representation of the Free Energy Function for Isotropic Thermoelastic Solids. Int. J. Solids Struct..

[23] Huang Z. (2014). A Novel Constitutive Formulation for Rubberlike Materials in Thermoelasticity. J. Appl. Mech..

[24] Yao X., Wang Z., Ma L., Miao Z., Su M., Han X., Yang J. (2022). Temperature Dependence of Rubber Hyper-Elasticity Based On Different Constitutive Models and their Prediction Ability. Polymers.

[25] Fu X., Wang Z., Ma L. (2021). Ability of Constitutive Models to Characterize the Temperature Dependence of Rubber Hyperelasticity and to Predict the Stress-Strain Behavior of Filled Rubber Under Different Defomation States. Polymers.

[26] Fu X., Wang Z., Ma L., Zou Z., Zhang Q., Guan X. (2020). Temperature-Dependence of Rubber Hyperelasticity Based On the Eight-Chain Model. Polymers.

[27] Huang Y., Li Y., Zhao H., Wen H. (2020). Research On Constitutive Models of Hydrogenated Nitrile Butadiene Rubber for Packer at Different Temperatures. J. Mech. Sci. Technol..

[28] Li X., Dong Y., Li Z., Xia Y. (2011). Experimental Study On the Temperature Dependence of Hyperelastic Behavior of Tire Rubbers Under Moderate Finite Deformation. Rubber Chem. Technol..

[29] Jin L., Xue Z., Wang Z., Li R., Liu J. (2023). Mechanical Response of the Sealing Packer Based On Two Rubber Materials at High Temperatures. Polym. Test..

[30] Ovalle Rodas C., Zaïri F., Naït-Abdelaziz M., Charrier P. (2015). Temperature and Filler Effects On the Relaxed Response of Filled Rubbers: Experimental Observations On a Carbon-Filled SBR and Constitutive Modeling. Int. J. Solids Struct..

[31] Li X., Bai T., Li Z., Liu L. (2016). Influence of the Temperature On the Hyper-Elastic Mechanical Behavior of Carbon Black Filled Natural Rubbers. Mech. Mater..

[32] Jiang D., Wang Z., Wang X. (2024). A General Hyperelastic Model for Rubber-Like Materials Incorporating Strain-Rate and Temperature. J. Elastomers Plast..

[33] Yan S., Jia D., Yu Y., Wang L., Qiu Y., Wan Q. (2020). Influence of Γ-Irradiation On Mechanical Behaviors of Poly Methyl-Vinyl Silicone Rubber Foams at Different Temperatures. Mech. Mater..

[34] Korba A.G., Kumar A., Barkey M. (2020). A Hyper-Elastic Thermal Aging Constitutive Model for Rubber-Like Materials. J. Elastomers Plast..

[35] Lou W., Xie C., Guan X. (2022). Thermal-Aging Constitutive Model for a Silicone Rubber Foam Under Compression. Polym. Degrad. Stab..

[36] Li Q., Xu Z.D., Tong Q.S., Dong Y.R., Xu Y.S., Lu Y. (2023). Mechanical Properties of Viscoelastic Materials Subjected to Thermal-Oxidative Aging: An Experimental and Theoretical Study. J. Appl. Polym. Sci..

[37] Li Q., Xu Z., Dong Y., He Z., He J., Yan X. (2023). Hyperelastic Hybrid Molecular Chain Model of Thermal-Oxidative Aging Viscoelastic Damping Materials Based on Physical–Chemical Process. J. Eng. Mech..

[38] Ha-Anh T., Vu-Khanh T. (2005). Prediction of Mechanical Properties of Polychloroprene During Thermo-Oxidative Aging. Polym. Test..

[39] Hu X., Yang X., Jiang X., Song K. (2024). Constitutive Model for Thermal-Oxygen-Aged EPDM Rubber Based on the Arrhenius Law. Polymers.

[40] Xie Z., Huang X., Zhang K., Yan S., Chen J., He R., Li J., Zhong W. (2025). Thermo-Oxidative Aging Effects on Hyperelastic Behavior of EPDM Rubber: A Constitutive Modeling Approach. Materials.

[41] Shakiba M., Najmeddine A. (2022). Physics-Based Constitutive Equation for Thermochemically Aged Elastomers Based On Crosslink Density Evolution. J. Mech. Mater. Struct..

[42] Zhi J., Wang Q., Zhang M., Zhou Z., Liu A., Jia Y. (2019). Coupled Analysis On Hyper-Viscoelastic Mechanical Behavior and Macromolecular Network Alteration of Rubber During Thermo-Oxidative Aging Process. Polymer.

[43] Li K., Zheng J., Zhi J., Zhang K. (2018). Aging Constitutive Model of Hydroxyl-Terminated Polybutadiene Coating in Solid Rocket Motor. Acta Astronaut..

[44] Du Y., Zheng J., Xiong C. (2020). Cross-Linking Density and Aging Constitutive Model of HTPB Coating Under Prestrain Thermal Accelerated Aging. Def. Technol..

[45] Liu Y., Zhang Q., Liu R., Chen M., Zhang C., Li X., Li W., Wang H. (2022). Compressive Stress-Hydrothermal Aging Behavior and Constitutive Model of Shield Tunnel EPDM Rubber Material. Constr. Build. Mater..

[46] Wang S., Xu J., Li H., Liu J., Zhou C. (2022). The Effect of Thermal Aging On the Mechanical Properties of Ethylene Propylene Diene Monomer Charge Coating. Mech. Time-Depend. Mater..

[47] Huang Y., Zhang F., Guo J., Xue J., Yi Z. (2014). Sealing Properties of EPDM Intercalation Composite Rings in High Temperature. Lubr. Eng..

[48] Gong C., Xie C., Zhu H., Ding W., Song J., Ge Y. (2024). Time-Varying Compressive Properties and Constitutive Model of EPDM Rubber Materials for Tunnel Gasketed Joint. Constr. Build. Mater..

[49] Celina M., Gillen K.T., Assink R.A. (2005). Accelerated Aging and Lifetime Prediction: Review of non-Arrhenius Behaviour Due to Two Competing Processes. Polym. Degrad. Stab..

[50] Cui Z., Liu W., Tan L., Sun G., Hu X. (2024). Evidence for non-Arrhenius Behavior of EPDM Rubber by Combining Arrhenius and Time–Temperature Superposition (TTS) Extrapolations. RSC Adv..

[51] Ben Hassine M., Naït-Abdelaziz M., Zaïri F., Colin X., Tourcher C., Marque G. (2014). Time to Failure Prediction in Rubber Components Subjected to Thermal Ageing: A Combined Approach Based upon the Intrinsic Defect Concept and the Fracture Mechanics. Mech. Mater..

[52] Lee J.H., Bae J.W., Kim J.S., Hwang T.J., Park S.D., Park S.H., Yeo T.M., Kim W., Jo N. (2011). Life-Time Prediction of a Chloroprene Rubber (CR) O-ring Using Intermittent Compression Stress Relaxation (CSR) and Time-Temperature Superposition (TTS) Principle. Macromol. Res..

[53] Bouaziz R., Ahose K.D., Lejeunes S., Eyheramendy D., Sosson F. (2019). Characterization and Modeling of Filled Rubber Submitted to Thermal Aging. Int. J. Solids Struct..

[54] Guo J., Xu P., Lv J., Han X., Sun Y., Hou D., Yuan Z., Li C. (2023). Ageing Behaviour and Molecular/Network Structure Evolution of EPDM/carbon Black Composites Under Compression and in Thermal-Oxidative Environments. Polym. Degrad. Stab..

